# The association of parental use of new tobacco products and combustible cigarettes with nausea and vomiting in pregnancy: a cross-sectional study

**DOI:** 10.1265/ehpm.25-00416

**Published:** 2026-03-28

**Authors:** Miki Akiyama, Takehiro Michikawa, Yuki Takeda, Sumito Nagasaki, Junya Sakuma, Keiko Asakura, Masahiko Nakata, Yuji Nishiwaki

**Affiliations:** 1 Department of Environmental and Occupational Health, Toho University Graduate School of Medicine, 5-21-16 Omori-nishi, Ota-ku, Tokyo 143-8540, Japan; 2 Department of Environmental and Occupational Health, School of Medicine, Toho University, 5-21-16 Omori-nishi, Ota-ku, Tokyo 143-8540, Japan; 3 Department of Community Well-being, School of Medicine, Toho University, 5-21-16 Omori-nishi, Ota-ku, Tokyo 143-8540, Japan; 4 Department of Obstetrics and Gynecology, Toho University Omori Medical Center, 6-11-1 Omori-nishi, Ota-ku, Tokyo 143-8541, Japan; 5 Department of Preventive Medicine, School of Medicine, Toho University, 5-21-16 Omori-nishi, Ota-ku, Tokyo 143-8540, Japan

**Keywords:** Nausea and vomiting in pregnancy (NVP), New tobacco products, Heated tobacco products, Electronic cigarettes (e-cigarettes), Combustible cigarettes

## Abstract

**Background:**

New tobacco products, including heated tobacco products and electronic cigarettes, are rapidly increasing in popularity among younger populations worldwide, particularly in Japan. Thus, clarifying the effects of both active and passive smoking of new tobacco products during pregnancy is an urgent public health concern. We focused on nausea and vomiting in pregnancy (NVP) which is traditionally and epidemiologically considered an indicator of healthy pregnancy progression. In this study, we classified maternal and paternal smoking status focussing on new tobacco product use and investigated its association with the absence of NVP.

**Methods:**

This cross-sectional study used control data from a case–control study designed to explore modifiable factors for anorectal malformations. The questionnaire survey was conducted within seven Japanese regions between December 2019 and March 2023, enrolling a total of 1,522 postpartum women. The study included 1,450 postpartum women who delivered singleton babies and provided complete information. The main outcome was the absence of NVP. The exposure was the smoking status of mothers and their partners, classified into four categories: dual users (combustible cigarettes and new tobacco products), new tobacco product-only users, combustible cigarette-only users, and non-smokers (reference). Logistic regression analysis was performed to estimate odds ratios (ORs) and 95% confidence intervals (CIs) for the absence of NVP.

**Results:**

Based on maternal smoking status, the absence of NVP was reported in 27.1% of dual users, 25.0% of new tobacco product–only users, 18.0% of combustible cigarette–only users, and 15.2% of non-smokers. Maternal dual use was associated with an increased OR of the absence of NVP (adjusted OR = 2.07, 95% CI: 1.02–4.22). After restricting to non-smoking mothers, the adjusted OR for paternal combustible cigarette smoking was 2.62 (95% CI: 1.25–5.50).

**Conclusion:**

Our main finding was the positive association between maternal dual use of new tobacco products and combustible cigarettes during pregnancy and the absence of NVP. This suggests that smoking cessation guidance during pregnancy, including new tobacco products, is particularly warranted.

**Trial registration:**

Not applicable.

**Supplementary information:**

The online version contains supplementary material available at https://doi.org/10.1265/ehpm.25-00416.

## Graphical Abstract

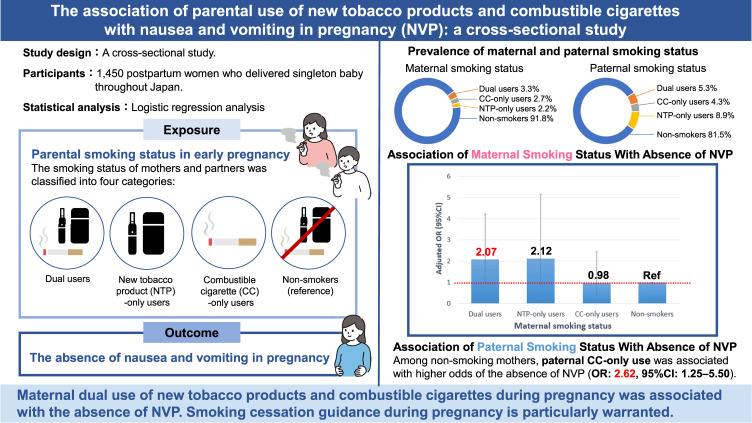

## Background

Nausea and vomiting in pregnancy (NVP) is the most common complication in early pregnancy, affecting 50–90% of pregnant women [1–3]. Typically, NVP begins at 6–8 weeks of gestation, peaks at approximately 9 weeks, and resolves by 12 weeks in most cases [2]. Although NVP imposes a burden on pregnant women, it has been hypothesised to protect both the mother and foetus from foodborne toxins and microorganisms [4], and/or to promote placental and foetal development through maternal undernutrition [1]. Indeed, NVP has traditionally been recognised as a sign of healthy and progressive pregnancy [5]. Consistent with this view, epidemiological studies have reported an association between NVP and a reduced risk of adverse foetal outcomes, including preterm birth and pregnancy loss [5, 6]. Therefore, NVP is likely a manifestation of normal physiological processes during pregnancy. Interestingly, although smoking is known to be associated with adverse perinatal outcomes [7], it has been suggested to reduce the occurrence of NVP [8, 9]. One possible mechanism is that maternal active smoking leads to abnormal placental development and affects the secretion of placental hormones associated with NVP [10, 11].

Globally and in Japan, the prevalence of smokers has been declining [12, 13]. However, the increasing use of new tobacco products, such as heated tobacco products and electronic cigarettes (e-cigarettes), among younger generations has become a significant public health concern [13–16]. Although these products differ from combustible cigarettes by producing aerosols through heating rather than combustion, their mainstream emissions also contain toxic substances [17, 18]. Recent studies have reported associations between the use of new tobacco products and adverse perinatal outcomes, including hypertensive disorders of pregnancy (HDP), preterm birth, and small for gestational age (SGA) [19–22]. Thus, we hypothesised that the use of new tobacco products is inversely associated with NVP, which is considered a sign of a healthy pregnancy. In addition, although new tobacco products do not produce secondhand smoke (i.e. emissions from the tip of a burning cigarette), harmful substances are present in the exhaled breath of smokers [23]. Therefore, concerns regarding the potential effects of passive smoking remain.

In this study, we aimed to explore whether active and passive smoking, including new tobacco products, during pregnancy is associated with the absence of NVP among pregnant women. These findings may offer insights into the mechanisms underlying the adverse perinatal effects of new tobacco products.

## Methods

### Participants

Participants in this cross-sectional study were postpartum women enrolled in a case-control study, which is designed to explore modifiable factors for anorectal malformations between December 2019 and March 2023. The main findings of this case-control study will be published separately (currently under preparation). Controls were recruited from 16 obstetric clinics and hospitals located in urban areas within seven Japanese regions, ensuring that they were drawn from the same source population as the cases identified across Japan. Specifically, two were in the Hokkaido and Tohoku regions, six were located in the Kanto region (three in Tokyo and three in other Kanto prefectures), one in the Tokai and Hokuriku regions, three in the Kinki region, two in the Chugoku and Shikoku regions, and two in the Kyushu and Okinawa regions. Postpartum women were eligible for inclusion if they delivered a baby without apparent congenital malformations at birth. During the period from childbirth to the 1-month check-up, obstetricians and/or midwives at each facility distributed a self-administered questionnaire to women, and responses were received by mail from 1,522 mothers (61% participation rate). Even if there were unanswered sections, we did not request that the participants provide responses to those to ensure complete data. After excluding 3 mothers with multiple births, 23 with missing maternal smoking information, 3 with missing paternal smoking information, 4 who did not respond to NVP, and 39 with incomplete covariate data, the final analytical sample comprised 1,450 mothers (Supplementary Fig. 1 in Additional file 1).

### Exposure

Maternal smoking status was used as the exposure variable for active smoking, and paternal smoking status was used as the exposure variable for passive smoking. Both pieces of information were obtained from the mothers.

Maternal smoking status was assessed using a questionnaire that asked about smoking habits during pregnancy: “Were you smoking cigarettes (burning and smoking) during pregnancy?” and “Were you smoking heated tobacco products or e-cigarettes during pregnancy?” Responses were categorised into four groups: “Yes, I smoked during pregnancy”, “I quit smoking when I found out about the current pregnancy”, “I quit smoking before pregnancy”, and “I have never smoked”. Mothers who responded “Yes, I smoked during pregnancy” or “I quit smoking when I found out about the current pregnancy” were defined as smokers. In addition, we collected detailed information on the mothers’ smoking habits during pregnancy. Mothers who smoked combustible cigarettes were asked to indicate the number of cigarettes smoked per day, categorised as 10 or fewer, 11–20, or 21 or more. Mothers who used new tobacco products during pregnancy were asked to specify the types of products used. Response options were: “Heated tobacco products or e-cigarettes containing nicotine”, E-cigarettes not containing nicotine”, and “E-cigarettes with unknown nicotine content”.

Paternal smoking status was assessed using the following yes/no questions: “Was the baby’s father smoking cigarettes (burning and smoking) at home when you found out about the current pregnancy?” and “Was the baby’s father smoking heated tobacco products or e-cigarettes at home when you found out about the current pregnancy?”

Based on these responses, mothers and their partners were classified into four groups by smoking status during pregnancy: (1) dual users (combustible cigarettes and new tobacco products); (2) new tobacco product–only users; (3) combustible cigarette–only users; and (4) non-smokers.

### Outcome

NVP was assessed with the question, “Did you experience nausea and vomiting during pregnancy?” with four response options: “No”, “Nausea only”, “Vomiting but able to eat”, and “Vomiting and unable to eat”. The outcome was the absence of NVP (corresponding to the “No” response), which was coded as “1”.

### Other variables

Data on maternal age, height, pre-pregnancy weight, maternal drinking during pregnancy, household income, parity, fertility treatment, and infant sex were collected using a questionnaire as potential confounders. Pre-pregnancy body mass index (BMI) was calculated from self-reported height and weight. Mothers who reported drinking during pregnancy were defined as those who responded either “I drank alcohol during my pregnancy” or “I stopped drinking alcohol when I found out I was pregnant”. The reason is that NVP typically develops during early pregnancy. Household income was selected from four predefined categories in the questionnaire (in million Japanese yen; <4, 4 to <8, 8 to <12, and ≥12). We also collected information on gestational age, birth length, birth weight, and pregnancy complications (HDP and gestational diabetes mellitus [GDM]).

### Statistical analysis

Continuous variables are presented as means (standard deviations), and categorical variables as percentages. Differences between groups were assessed using analysis of variance for continuous variables and the chi-squared test for categorical variables.

The association between maternal and paternal smoking status (independent variables) and the absence of NVP (dependent variable) was investigated using logistic regression analysis. We estimated the odds ratios (ORs) and 95% confidence intervals (CIs) for the absence of NVP across each smoking category, using the non-smoker group as the reference. In adjusted model, we adjusted for maternal age, pre-pregnancy BMI, alcohol consumption during pregnancy (yes or no), household income (in million Japanese yen; <4, 4 to <8, 8 to <12, and ≥12), parity (primipara or multipara), fertility treatment (yes or no), and infant sex (male or female). Additionally, when maternal smoking status was considered an exposure factor, paternal smoking status was included as a confounder. Conversely, when paternal smoking status was considered an exposure factor, maternal smoking status was included as a confounder. As a sensitivity analysis 1, we excluded 19 mothers who answered “E-cigarettes not containing nicotine” or “E-cigarettes with unknown nicotine content,” given that nicotine is known to have adverse effects on pregnancy [10, 24]. In addition, we conducted analyses excluding mothers who smoked during pregnancy when paternal smoking status was treated as the exposure, to minimise the potential confounding of maternal smoking (sensitivity analysis 2).

All analyses were performed using Stata version 16 for Windows (Stata Corporation, College Station, TX, USA).

## Results

The characteristics of the 1,450 mothers according to smoking status are presented in Table 1. The majority of mothers were non-smokers (91.8%), followed by dual users (3.3%), combustible cigarette-only users (2.7%), and new tobacco product-only users (2.2%). Some characteristics, such as maternal age at delivery, alcohol consumption status, pre-pregnancy BMI, and household income, differed between the groups. Background information based on paternal smoking status is presented in Supplementary Table 1 (Additional file 1).

**Table 1:** Characteristics of 1,450 mothers

	**Number (%) or mean ± SD**					***p* value**

	**Total**	**Maternal smoking status during pregnancy**				

		**Dual users**	**NTP-only users**	**CC-only users**	**Non-smokers**	

	**n = 1450**	**n = 48**	**n = 32**	**n = 39**	**n = 1331**	
Maternal age at delivery (years)	32.6 ± 4.6	30.1 ± 5.1	31.8 ± 4.8	30.2 ± 4.6	32.7 ± 4.5	<0.01
Primiparity	657 (45.3)	23 (47.9)	11 (34.4)	20 (51.3)	603 (45.3)	0.53
Fertility treatment	276 (19.0)	4 (8.3)	4 (12.5)	6 (15.4)	262 (19.7)	0.16
Alcohol drinking during pregnancy	628 (43.3)	40 (83.3)	23 (71.9)	26 (66.7)	539 (40.5)	<0.01
Pre-pregnancy body mass index (kg/m^2^)	21.2 ± 3.1	22.5 ± 4.1	21.5 ± 3.9	21.6 ± 3.6	21.1 ± 3.0	0.01
<18.5	216 (14.9)	7 (14.6)	7 (21.9)	5 (12.8)	197 (14.8)	0.01
18.5–24.9	1083 (74.7)	29 (60.4)	19 (59.4)	30 (76.9)	1005 (75.5)	
≥25.0	151 (10.4)	12 (25.0)	6 (18.8)	4 (10.3)	129 (9.7)	
Infant’s sex (male)	759 (52.3)	24 (50.0)	20 (62.5)	22 (56.4)	693 (52.1)	0.63
Household income (million JPY/year)						
<4	166 (11.5)	19 (39.6)	7 (21.9)	12 (30.8)	128 (9.6)	<0.01
≥4 to <8	729 (50.3)	18 (37.5)	17 (53.1)	20 (51.3)	674 (50.6)	
≥8 to <12	402 (27.7)	8 (16.7)	6 (18.8)	7 (18.0)	381 (28.6)	
≥12	153 (10.6)	3 (6.3)	2 (6.3)	0 (0.0)	148 (11.1)	
Paternal age at delivery (years)*	34.4 ± 5.7	32.3 ± 7.0	34.1 ± 5.8	32.0 ± 6.5	34.5 ± 5.6	<0.01
Paternal smoking status during pregnancy						
Dual users	77 (5.3)	14 (29.2)	2 (6.3)	4 (10.3)	57 (4.3)	<0.01
NTP-only users	129 (8.9)	10 (20.8)	21 (65.6)	2 (5.1)	96 (7.2)	
CC-only users	62 (4.3)	5 (10.4)	2 (6.3)	17 (43.6)	38 (2.9)	
Non-smokers	1182 (81.5)	19 (39.6)	7 (21.9)	16 (41.0)	1140 (85.7)	

CC: combustible cigarette, NTP: new tobacco product, SD: standard deviation

*Several participants did not answer the question (one in the dual user group and one in the non-smoker group).

Detailed information on maternal smoking status is presented in Table 2. Six combustible cigarette-only users (15.4%) and eight new tobacco product-only users (25.0%) continued smoking during pregnancy. Seventeen dual users (39.6%) and 9 combustible cigarette-only users (25.7%) reported smoking 11 or more cigarettes per day. Thirty-six dual users (78.3%) and 22 new tobacco product-only users (71.0%) reported using heated tobacco products or e-cigarettes containing nicotine.

**Table 2:** Detailed information on maternal smoking status during pregnancy (n = 1,450)

	**Number (%)**			

	**Maternal smoking status during pregnancy**			

	**Dual users**	**NTP-only users**	**CC-only users**	**Non-smokers**

	**n = 48**	**n = 32**	**n = 39**	**n = 1331**
Maternal smoking history related to combustible cigarettes				
Current smoker during pregnancy	5 (10.4)	0 (0.0)	6 (15.4)	0 (0.0)
Former smoker (Quit after finding out pregnancy)	43 (89.6)	0 (0.0)	33 (84.6)	0 (0.0)
Former smoker (Quit before pregnancy)	0 (0.0)	27 (84.4)	0 (0.0)	158 (11.9)
Never smoker	0 (0.0)	5 (15.6)	0 (0.0)	1173 (88.1)

Number of combustible cigarettes per day during pregnancy*				
≥1 to ≤10	26 (60.5)	—	26 (74.3)	—
≥11 to ≤20	15 (34.9)	—	9 (25.7)	—
≥21	2 (4.7)	—	0 (0.0)	—

Maternal smoking history related to new tobacco products				
Current smoker during pregnancy	7 (14.6)	8 (25.0)	0 (0.0)	0 (0.0)
Former smoker (Quit after finding out pregnancy)	41 (85.4)	24 (75.0)	0 (0.0)	0 (0.0)
Former smoker (Quit before pregnancy)	0 (0.0)	0 (0.0)	7 (18.0)	51 (3.8)
Never smoker	0 (0.0)	0 (0.0)	32 (82.1)	1280 (96.2)

Type of new tobacco products smoked during pregnancy**				
Heated tobacco products or e-cigarettes containing nicotine	36 (78.3)	22 (71.0)	—	—
E-cigarettes not containing nicotine	6 (13.0)	4 (12.9)	—	—
E-cigarettes with unknown nicotine content	4 (8.7)	5 (16.1)	—	—

CC: combustible cigarette, E-cigarettes: electronic cigarettes, NTP: new tobacco product

*Several participants did not answer the question (five in the dual user group and four in the CC only user group).

**Several participants did not answer the question (two in the dual user group and one in the NTP only user group).

The association between maternal smoking and the absence of NVP is shown in Table 3. The overall prevalence of the absence of NVP was 15.9%. The prevalence by smoking status was 27.1% among dual users, 25.0% among new tobacco product-only users, 18.0% among combustible cigarette-only users, and 15.2% among non-smokers. After adjusting for several confounding factors (Adjusted model), mothers who were dual users had an increased OR for the absence of NVP (OR = 2.07, 95% CI: 1.02–4.22). A similar OR was observed (OR = 2.12, 95% CI: 0.87–5.14) among new tobacco product-only users, although it was not statistically significant. In sensitivity analysis 1, which was restricted to mothers who used nicotine-containing new tobacco products, a significant association was again observed among dual users (OR = 2.30, 95% CI: 1.05–5.03).

**Table 3:** Association between maternal smoking during pregnancy and the absence of nausea and vomiting in pregnancy

	**Dual users**	**NTP-only users**	**CC-only users**	**Non-smokers**
Main analysis				
Prevalence n/N (%)	13/48 (27.1)	8/32 (25.0)	7/39 (18.0)	202/1331 (15.2)
Crude, OR (95% CI)	2.08 (1.08–3.99)	1.86 (0.83–4.20)	1.22 (0.53–2.81)	Ref
Adjusted model*, OR (95% CI)	2.07 (1.02–4.22)	2.12 (0.87–5.14)	0.98 (0.40–2.44)	Ref

Sensitivity analysis 1**				
Prevalence n/N (%)	11/38 (29.0)	6/23 (26.1)	7/39 (18.0)	202/1331 (15.2)
Adjusted model*, OR (95% CI)	2.30 (1.05–5.03)	2.20 (0.79–6.07)	0.99 (0.40–2.46)	Ref

CC: combustible cigarette, CI: confidence interval, NTP: new tobacco product, OR: odds ratio

*Adjusted for maternal age, pre-pregnancy body mass index (BMI), maternal drinking during pregnancy, household income, infant’s sex, parity, fertility treatment, and paternal smoking status.

**Sensitivity analysis 1 was conducted among 1,431 participants after excluding 19 mothers who used e-cigarettes not containing nicotine or e-cigarettes with unknown nicotine content.

The association between paternal smoking and the absence of NVP is shown in Table 4. The prevalence of absence of NVP by smoking status was 18.2% among dual users, 14.7% among new tobacco product-only users, 22.6% among combustible cigarette-only users, and 15.5% among non-smokers. No significant association was observed between paternal smoking status and the absence of NVP in the adjusted model and sensitivity analysis 1. After excluding mothers who smoked during pregnancy (background information of the participants in this sensitivity analysis 2 is presented in Supplementary Table 2 [Additional File 1]), the adjusted OR of the absence of NVP was 2.62 (95% CI: 1.25–5.50) among combustible cigarette–only users.

**Table 4:** Association between paternal smoking during pregnancy and the absence of nausea and vomiting in pregnancy

	**Dual users**	**NTP-only users**	**CC-only users**	**Non-smokers**
Main analysis				
Prevalence n/N (%)	14/77 (18.2)	19/129 (14.7)	14/62 (22.6)	183/1182 (15.5)
Crude, OR (95% CI)	1.21 (0.67–2.21)	0.94 (0.57–1.57)	1.59 (0.86–2.95)	Ref
Adjusted model*, OR (95% CI)	1.04 (0.55–1.98)	0.77 (0.44–1.35)	1.54 (0.79–3.03)	Ref

Sensitivity analysis 1**				
Prevalence n/N (%)	13/74 (17.6)	18/122 (14.8)	14/61 (23.0)	181/1174 (15.4)
Adjusted model*, OR (95% CI)	0.99 (0.51–1.93)	0.79 (0.45–1.40)	1.58 (0.80–3.13)	Ref

Sensitivity analysis 2***				
Prevalence n/N (%)	9/57 (15.8)	13/96 (13.5)	11/38 (29.0)	169/1140 (14.8)
Adjusted model****, OR (95% CI)	1.08 (0.51–2.29)	0.89 (0.48–1.65)	2.62 (1.25–5.50)	Ref

CC: combustible cigarette, CI: confidence interval, NTP: new tobacco product, OR: odds ratio

*Adjusted for maternal age, pre-pregnancy body mass index (BMI), maternal drinking during pregnancy, household income, infant’s sex, parity, fertility treatment, and maternal smoking status.

**Sensitivity analysis 1 was conducted among 1,431 participants after excluding 19 mothers who used e-cigarettes not containing nicotine or e-cigarettes with unknown nicotine content.

***Sensitivity analysis 2 was conducted among 1,331 participants after excluding 119 mothers who smoked during pregnancy.

****Adjusted for maternal age, pre-pregnancy BMI, maternal drinking during pregnancy, household income, infant’s sex, parity, and fertility treatment.

## Discussion

We found a positive association between maternal dual use of new tobacco products and combustible cigarettes and the absence of NVP. Interestingly, maternal use of new tobacco products alone tended to increase the odds of absence of NVP, and this tendency persisted in sensitivity analysis. New tobacco products produce aerosols containing harmful substances, such as nicotine, carbonyls, tobacco-specific nitrosamines (TSNAs), reactive oxygen species, and heavy metals [17, 25–29]. Furthermore, in recent years, the combined use of new tobacco products and combustible cigarettes was reported to be as harmful as the use of combustible cigarettes alone [30]. Thus, our finding is not surprising.

The onset of NVP has traditionally and epidemiologically been recognised as a sign of a healthy and progressing pregnancy [5, 6]. However, the mechanisms underlying the onset of NVP are not fully understood. One possible explanation involves the various endocrine factors secreted by the placenta, including human chorionic gonadotropin (hCG), oestrogen, and progesterone [31]. Recently, growth differentiation factor 15 (GDF15), which has been proposed as a key factor in the development of NVP, has been suggested to originate from the foetal-placental unit, including the placenta [32]. As increases in the levels of these hormones are thought to contribute to the onset of NVP, the placenta plays a central role in its development.

Previous research has clearly demonstrated that combustible cigarettes adversely affect placental development [10, 24]. Holloway et al. suggested that nicotine exposure during pregnancy inhibits trophoblast differentiation and invasion, increases placental hypoxia, and disrupts vascular formation in the labyrinth zone of the placenta [24]. Earlier studies have reported that smokers exhibit lower levels of hCG and pregnancy-associated plasma protein A, both secreted from the placenta, than non-smokers [11, 33]. Furthermore, the levels of inhibin A—an endocrine marker associated with HDP and considered an indicator of trophoblast dysfunction [34]—were reported to be higher in smokers than in non-smokers [33]. These findings suggest that smoking impairs placental development and affects the secretion of key placental hormones, which may contribute to the absence of NVP. Previous epidemiological studies have reported positive associations between maternal use of new tobacco products and placenta-mediated complications [35], including HDP, preterm birth, and SGA [19–22]. Consistent with these findings, our results suggest that maternal use of new tobacco products, particularly dual use, may be associated with impaired placental development. Moreover, in this study, among dual users, the prevalence of GDM—which is thought to be associated with placental function [36]—was higher in those without NVP than in those with NVP (Supplementary Table 3 in Additional file 1). Further research, including analysis of placental tissue and endocrine markers, is warranted to clarify the effects of new tobacco products on placental development and function. Also, although NVP imposes a burden on pregnant women, it should be emphasised that NVP has been associated with favourable foetal outcomes and has been regarded as a sign of a normal and healthy pregnancy course. Therefore, the observed association between maternal dual use and the absence of NVP in this study should not be interpreted as indicating any beneficial effect of smoking during pregnancy. Smoking, including the use of new tobacco products, adversely affects pregnancy and foetal development. Accordingly, smoking cessation during pregnancy remains essential.

In this study, no association was found between pregnant women who smoked only combustible cigarettes and the absence of NVP. The results seem to contradict our earlier explanation, as well as previous epidemiological findings that maternal active smoking of combustible cigarettes during pregnancy reduced the occurrence of NVP [8, 9]. One possible explanation is that the well-known adverse effects of combustible cigarettes on pregnancy may have encouraged smoking cessation or reduced cigarette consumption [37]. In fact, Table 2 shows that combustible cigarette-only users were less likely to continue smoking during pregnancy than new tobacco product-only users. In addition, combustible cigarette users tended to smoke fewer cigarettes than dual users. These behaviours of combustible cigarette-only users may explain the absence of an association.

This study found no significant association between paternal smoking at home during early pregnancy and the absence of NVP in the main analysis. However, when mothers who smoked during pregnancy were excluded, a positive association was observed among combustible cigarette–only users. The relatively large effect size (OR = 2.62) of passive smoking from combustible cigarettes is consistent with previous evidence that passive exposure to combustible cigarettes adversely affects maternal and foetal health [38, 39]. By contrast, no positive association was observed between the use of new tobacco products and the absence of NVP, both among dual users and new tobacco product–only users. Unlike combustible cigarettes, new tobacco products do not generate secondhand smoke; therefore, the pattern and intensity of passive smoking exposure may differ. However, we were unable to directly assess differences in exposure levels between combustible cigarette smoke and aerosols emitted from new tobacco products. At the same time, passive exposure itself cannot be considered absent, as exhaled breath from users of new tobacco products contains harmful substances [23]. Consistent with this concern, our previous study reported an association between paternal dual use of new tobacco products and combustible cigarettes and HDP [40]. One possible explanation for the lack of increased odds among dual users in the present study is the difference in outcome timing. Whereas HDP typically develop after 20 weeks of gestation [40], this study focused on NVP, which occurs predominantly in early pregnancy. Previous studies have suggested that users of new tobacco products may have difficulty quitting smoking [41]. Given that HDP is influenced by exposure extending beyond early pregnancy, differences in the timing of outcome onset and the relevant exposure period may partly explain the inconsistency in findings.

The main strength of our study is that, to the best of our knowledge, this is the first study to examine the potential effects of new tobacco products on NVP in Japan, the first country in the world to market heated tobacco products [42]. Therefore, this study provides valuable information for other countries in which the use of new tobacco products is increasing. Another strength is that we successfully obtained information on the smoking status of pregnant women and their partners, and accounted for both active and passive smoking. We also presented information on the characteristics of mothers and partners according to their smoking status. This information may be beneficial for future smoking cessation counselling. Finally, the study population can be considered to have reasonable generalisability. According to the 2023 National Health and Nutrition Survey in Japan, smoking prevalence among women aged 20–39 years ranged from 5.2% to 8.7%; the prevalence among mothers in the present study (8.2%) was therefore within the range reported for the general population [13]. In addition, the distribution of maternal smoking categories in the present study was comparable to that reported in previous Japanese study [20], although differences in exposure definitions may partly explain the slightly higher proportion of new tobacco product users observed in this study. Moreover, as shown in Supplementary Table 4 in Additional file 1, the key demographic characteristics of the study population were also comparable to those reported in previous large-scale cohort studies [43]. Together, these findings support the representativeness of the study population among pregnant women in Japan.

However, this study has some limitations. First, NVP was determined based on mothers’ self-reports without the use of objective indicators, such as rate of weight loss, clinical signs of dehydration, or urinary ketones [2]. Although there may be individual differences in the severity of NVP, symptoms such as nausea and vomiting are generally readily recognisable. Furthermore, since the prevalence of NVP in this study was consistent with previous reports [1, 2], we believe that our outcome assessment is reasonable. Secondly, this study did not distinguish between e-cigarettes and heated tobacco products. In Japan, heated tobacco products are often referred to as e-cigarettes [44]. In addressing this issue, we asked mothers who reported using new tobacco products during pregnancy whether the products contained nicotine. As shown in Table 2, more than 70% of participants in both the dual-use and new tobacco product-only groups reported using nicotine-containing products. In Japan, the sale of nicotine-containing e-cigarettes has been prohibited under the Pharmaceutical Affairs Law since 2010 [45]; therefore, it is presumed that most mothers use heated tobacco products. Third, as an indicator of passive smoking, we obtained information on only paternal smoking at home during early pregnancy from mothers. Thus, we could not exclude the effect of unmeasured and residual confounding factors, such as smoking by other cohabiting family members and passive smoking outside the home. However, because this study focused on NVP, we considered it sufficient to ask about paternal smoking during early pregnancy, and obtaining this information from mothers was reasonable as an indicator of passive smoking. Fourth, the participation rate in this study was not so high (approximately 60%), and comparisons between participants and non-participants were not possible. Therefore, the possibility of selection bias cannot be ruled out. In addition, exposure information was obtained from postpartum women, and recall in early pregnancy may have been imperfect; however, to minimise recall bias, questionnaires were distributed between delivery and the 1-month postnatal check-up. Finally, because of the cross-sectional nature of this study, we could not demonstrate a causal relationship between smoking and the absence of NVP.

## Conclusions

Maternal dual use of new tobacco products and combustible cigarettes during pregnancy was positively associated with the absence of NVP. Among mothers who were non-smokers, paternal combustible cigarette smoking at home during early pregnancy was associated with higher odds of the absence of NVP. Although it is not possible to draw definitive conclusions on the adverse effects of new tobacco products on perinatal outcomes, our findings suggest that smoking may adversely affect placental development. Accordingly, smoking cessation guidance during pregnancy, including new tobacco products, is particularly warranted.

## Abbreviations

BMI: body mass index
CC: combustible cigarette
e-cigarettes: electronic cigarettes
GDF15: growth differentiation factor 15
GDM: gestational diabetes mellitus
hCG: human chorionic gonadotropin
HDP: hypertensive disorders of pregnancy
NTP: new tobacco product
NVP: nausea and vomiting in pregnancy
SGA: small for gestational age
TSNAs: tobacco-specific nitrosamines

## Supplementary information

**Additional file 1:** **Supplementary Figure 1.** Flowchart of participant selection. **Supplementary Table 1.** Characteristics of 1,450 mothers according to their paternal smoking status during pregnancy. **Supplementary Table 2.** Characteristics of 1,331 mothers who did not smoke during pregnancy according to paternal smoking status during pregnancy (sensitivity analysis 2). **Supplementary Table 3.** Smoking status stratified by prevalence of pregnancy complications, including preterm birth, low birth weight, small for gestational age, hypertensive disorders of pregnancy, and gestational diabetes mellitus, between women with and without nausea and vomiting in pregnancy. **Supplementary Table 4.** Comparison of demographic characteristics between the present study population and populations in previous studies.

## Declarations

### Availability of data and material

Data cannot be shared for privacy or ethical reasons.
