# Supplementary material for: The association of parental use of new tobacco products and combustible cigarettes with nausea and vomiting in pregnancy: a cross-sectional study
Source: Environ Health Prev Med. 2026 Mar 28;31:24. doi: 10.1265/ehpm.25-00416 (PMC13057909; doi:10.1265/ehpm.25-00416)
Supplement: Supplementary file 1 — Additional file 1: Supplementary Figure 1. Flowchart of participant selection. Supplementary Table 1. Characteristics of 1,450 mothers according to their paternal smoking status during pregnancy. Supplementary Table 2. Characteristics of 1,331 mothers who did not smoke during pregnancy according to paternal smoking status during pregnancy (sensitivity analysis 2). Supplementary Table 3. Smoking status stratified by prevalence of pregnancy complications, including preterm birth, low birth weight, small for gestational age, hypertensive disorders of pregnancy, and gestational diabetes mellitus, between women with and without nausea and vomiting in pregnancy. Supplementary Table 4. Comparison of demographic characteristics between the present study population and populations in previous studies. [file ehpm-31-024-s001.docx]

**Additional file 1**

**Supplementary figure and tables**

Supplementary Figure 1. Flowchart of participant selection


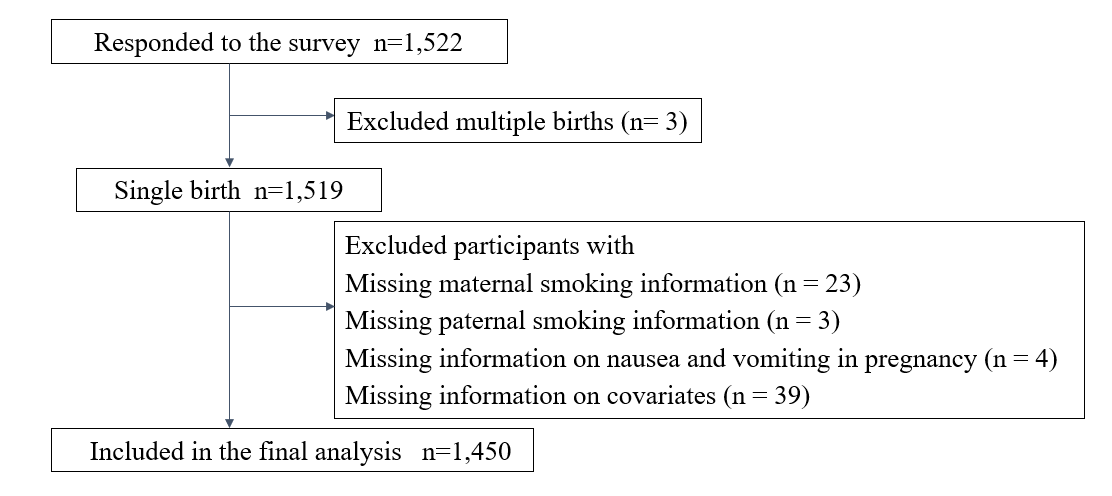


Supplementary Table 1. Characteristics of 1,450 mothers according to their paternal smoking status during pregnancy

|  | Number (%) or mean ± SD | | | | | *p* value |
| --- | --- | --- | --- | --- | --- | --- |
|  | Total | Paternal smoking status during pregnancy | | | |  |
|  |  | Dual users | NTP-only users | CC-only users | Non-smokers |  |
|  | n = 1450 | n = 77 | n = 129 | n = 62 | n = 1182 |  |
| Maternal age at delivery (years) | 32.6 ± 4.6 | 30.0 ± 5.4 | 32.6 ± 4.4 | 31.3 ± 5.0 | 32.8 ± 4.5 | <0.01 |
| Primiparity | 657 (45.3) | 41 (53.3) | 55 (42.6) | 28 (45.2) | 533 (45.1) | 0.50 |
| Fertility treatment | 276 (19.0) | 4 (5.2) | 27 (20.9) | 10 (16.1) | 235 (19.9) | 0.01 |
| Alcohol drinking during pregnancy | 628 (43.3) | 44 (57.1) | 72 (55.8) | 32 (51.6) | 480 (40.6) | <0.01 |
| Pre-pregnancy body mass index (kg/m^2^) | 21.2 ± 3.1 | 21.2 ± 2.8 | 21.2 ± 3.3 | 21.4 ± 2.9 | 21.1 ± 3.1 | 0.93 |
| < 18.5 | 216 (14.9) | 10 (13.0) | 24 (18.6) | 8 (12.9) | 174 (14.7) | 0.52 |
| 18.5–24.9 | 1083 (74.7) | 61 (79.2) | 88 (68.2) | 45 (72.6) | 889 (75.2) |  |
| ≥ 25.0 | 151 (10.4) | 6 (7.8) | 17 (13.2) | 9 (14.5) | 119 (10.1) |  |
| Infant’s sex (male) | 759 (52.3) | 41 (53.3) | 66 (51.2) | 31 (50.0) | 621 (52.5) | 0.97 |
| Household income (million JPY/year) |  |  |  |  |  |  |
| < 4 | 166 (11.5) | 25 (32.5) | 21 (16.3) | 15 (24.2) | 105 (8.9) | <0.01 |
| ≥ 4 to < 8 | 729 (50.3) | 40 (52.0) | 69 (53.5) | 38 (61.3) | 582 (49.2) |  |
| ≥ 8 to < 12 | 402 (27.7) | 9 (11.7) | 30 (23.3) | 8 (12.9) | 355 (30.0) |  |
| ≥ 12 | 153 (10.6) | 3 (3.9) | 9 (7.0) | 1 (1.6) | 140 (11.8) |  |
| Paternal age at delivery (years)* | 34.4 ± 5.7 | 32.4 ± 7.4 | 34.4 ± 5.3 | 33.9 ± 5.7 | 34.5 ± 5.6 | 0.01 |
| Maternal smoking status during pregnancy |  |  |  |  |  |  |
| Dual users | 48 (3.3) | 14 (18.2) | 10 (7.8) | 5 (8.1) | 19 (1.6) | <0.01 |
| NTP-only users | 32 (2.2) | 2 (2.6) | 21 (16.3) | 2 (3.2) | 7 (0.6) |  |
| CC-only users | 39 (2.7) | 4 (5.2) | 2 (1.6) | 17 (27.4) | 16 (1.4) |  |
| Non-smokers | 1331 (91.8) | 57 (74.0) | 96 (74.4) | 38 (61.3) | 1140 (96.5) |  |
| CC: combustible cigarette, NTP: new tobacco product, SD: standard deviation  *Several participants did not answer the question (one in the dual user group and one in the non-smokers group). | | | | | | |

Supplementary Table 2. Characteristics of 1,331 mothers who did not smoke during pregnancy according to paternal smoking status during pregnancy (sensitivity analysis 2)

|  | Number (%) or mean ± SD | | | | | *p* value |
| --- | --- | --- | --- | --- | --- | --- |
|  | Total | Paternal smoking status during pregnancy | | | |  |
|  |  | Dual users | NTP-only users | CC-only users | Non-smokers |  |
|  | n = 1331 | n = 57 | n = 96 | n = 38 | n = 1140 |  |
| Maternal age at delivery (years) | 32.7 ± 4.5 | 30.4 ± 5.2 | 33.1 ± 4.4 | 31.8 ± 5.1 | 32.9 ± 4.5 | <0.01 |
| Primiparity | 603 (45.3) | 29 (50.9) | 43 (44.8) | 16 (42.1) | 515 (45.2) | 0.83 |
| Fertility treatment | 262 (19.7) | 4 (7.0) | 23 (24.0) | 8 (21.1) | 227 (19.9) | 0.07 |
| Alcohol drinking during pregnancy | 539 (40.5) | 29 (50.9) | 46 (47.9) | 16 (42.1) | 448 (39.3) | 0.14 |
| Pre-pregnancy body mass index (kg/m^2^) | 21.1 ± 3.0 | 20.9 ± 2.4 | 21.2 ± 3.3 | 21.9 ± 2.9 | 21.1 ± 3.0 | 0.34 |
| < 18.5 | 197 (14.8) | 7 (12.3) | 19 (19.8) | 3 (7.9) | 168 (14.7) | 0.26 |
| 18.5–24.9 | 1005 (75.5) | 47 (82.5) | 65 (67.7) | 29 (76.3) | 864 (75.8) |  |
| ≥ 25.0 | 129 (9.7) | 3 (5.3) | 12 (12.5) | 6 (15.8) | 108 (9.5) |  |
| Infant’s sex (male) | 693 (52.1) | 29 (50.9) | 48 (50.0) | 19 (50.0) | 597 (52.4) | 0.96 |
| Household income (million JPY/year) |  |  |  |  |  |  |
| < 4 | 128 (9.6) | 14 (24.6) | 9 (9.4) | 9 (23.7) | 96 (8.4) | <0.01 |
| ≥ 4 to < 8 | 674 (50.6) | 34 (59.7) | 53 (55.2) | 25 (65.8) | 562 (49.3) |  |
| ≥ 8 to < 12 | 381 (28.6) | 6 (10.5) | 25 (26.0) | 3 (7.9) | 347 (30.4) |  |
| ≥ 12 | 148 (11.1) | 3 (5.3) | 9 (9.4) | 1 (2.6) | 135 (11.8) |  |
| Paternal age at delivery (years)* | 34.5 ± 5.6 | 33.6 ± 7.2 | 34.4 ± 5.3 | 35.1 ± 5.4 | 34.6 ± 5.5 | 0.51 |
| CC: combustible cigarette, NTP: new tobacco product, SD: standard deviation  *Several participants did not answer the question (one in the dual user group and one in the non-smokers group). | | | | | | |

Supplementary table 3. Smoking status stratified by prevalence of pregnancy complications, including preterm birth, low birth weight, small for gestational age, hypertensive disorders of pregnancy, and gestational diabetes mellitus, between women with and without nausea and vomiting in pregnancy

|  | Total | Preterm birth* | | Low birth weight** | | SGA *** | | HDP | | GDM | |
| --- | --- | --- | --- | --- | --- | --- | --- | --- | --- | --- | --- |
|  | n (%) | n (%) | p-value by Fisher’s exact test | n (%) | p-value by Fisher’s exact test | n (%) | p-value by Fisher’s exact test | n (%) | p-value by Fisher’s exact test | n (%) | p-value by Fisher’s exact test |
| Dual users | | | | | | | | | | | |
| NVP | 35 (100.0) | 2 (5.7) | 0.27 | 2 (5.7) | 0.29 | 0 (0.0) | NA**** | 3 (8.6) | 0.55 | 4 (11.4) | 0.05 |
| No NVP | 13 (100.0) | 2 (16.7) |  | 2 (15.4) |  | 0 (0.0) |  | 0 (0.0) |  | 5 (38.5) |  |
| NTP-only users | | | | | | | | | | | |
| NVP | 24 (100.0) | 0 (0.0) | NA**** | 3 (12.5) | 0.56 | 0 (0.0) | 0.25 | 1 (4.2) | 1.00 | 1 (4.2) | 1.00 |
| No NVP | 8 (100.0) | 0 (0.0) |  | 0 (0.0) |  | 1 (12.5) |  | 0 (0.0) |  | 0 (0.0) |  |
| CC-only users | | | | | | | | | | | |
| NVP | 32 (100.0) | 1 (3.1) | 1.00 | 0 (0.0) | NA**** | 1 (3.1) | 1.00 | 0 (0.0) | NA**** | 1 (3.1) | 0.33 |
| No NVP | 7 (100.0) | 0 (0.0) |  | 0 (0.0) |  | 0 (0.0) |  | 0 (0.0) |  | 1 (14.3) |  |
| Non-smokers | | | | | | | | | | | |
| NVP | 1129 (100.0) | 31 (2.8) | 0.82 | 61 (5.4) | 0.03 | 28 (2.5) | 0.81 | 43 (3.8) | 0.30 | 72 (6.4) | 0.53 |
| No NVP | 202 (100.0) | 6 (3.0) |  | 4 (2.0) |  | 4 (2.0) |  | 4 (2.0) |  | 10 (5.0) |  |
| CC: combustible cigarette, NA: not available, NTP: new tobacco product, SGA: small for gestational age,  HDP: hypertensive disorders of pregnancy, GDM: gestational diabetes mellitus, NVP: nausea and vomiting in pregnancy  * Preterm birth was defined as delivery before 37 weeks of gestation [Tucker J, McGuire W. BMJ. 2004 Sep 18;329(7467):675-8]. Several participants did not answer the question (one in the dual user group and two in the non-smoker group).  ** Low birth weight was defined as a birth weight of less than 2,500 g, in accordance with the World Health Organization definition.  *** SGA was defined as being born with a birth weight and/or birth length below −2 standard deviation scores for gestational age [Hokken-Koelega, et al. Endocr Rev, 2023. 44(3): 539-565]. Several participants did not answer the question (two in the dual user group and 13 in the non-smoker group).  **** NA indicates that p-values could not be calculated due to no applicable cases. | | | | | | | | | | | |

Supplementary table 4. Comparison of demographic characteristics between the present study population and populations in previous studies

|  | This study | Hokkaido Cohort | TMM BirThree Cohort^1^ | HBC Study^2^ | BOSHI Cohort^3^ |
| --- | --- | --- | --- | --- | --- |
|  | n = 1,450 | n = 16,733 | n = 8,979 | n = 1,258 | n = 1,249 |
| Recruitment period | 2019~2023 | 2002~2012 | 2013~2017 | 2007~2011 | 2006~2010 |
| Study area | Throughout Japan | Hokkaido prefecture | Miyagi and Iwate prefectures | Hamamatsu city, Shizuoka | Sendai city, Miyagi |
| Maternal age at delivery (years) | 32.6 ± 4.6 | 30.3 ± 4.8 | 32.0 ± 4.8 | 31.5 ± 5.1 | 31.2 ± 4.9 |
| Pre-pregnancy body mass index (kg/m^2^) | 21.2 ± 3.1 | 21.1 ± 3.3 | 21.3 ± 3.3 | 21.0 ± 3.3 | 21.7 ± 3.4 |
| Primiparity | 657 (45.3) | 7,093 (42.4) | 2,940 (32.7) | 626 (49.8) | 717 (57.4) |
| Infant’s sex (male) | 759 (52.3) | 9,284 (55.4) | 4,666 (52.0) | 647 (51.4) | 637 (51.0) |

This table was created for comparison with the general population, based on Table 1 in Morisaki N, et al., Journal of Epidemiology, 2023 (reference 43 in the main text).

1. TMM BirThree: Tohoku Medical Megabank Project Birth and Three-Generation Cohort Study.
2. HBC Study: Hamamatsu Birth Cohort for Mothers and Children Study.
3. BOSHI Cohort: Babies and their Parents’ Longitudinal Observation in Suzuki Memorial Hospital in Intrauterine Period Study.
